# IoT2Wallets: A Blockchain-Based Access Control and Consent-Auditing Architecture for Patient-Controlled Health IoT Data Integration

**DOI:** 10.3390/s26206658

**Published:** 2026-10-21

**Authors:** João C. Ferreira, Marco Esperança, Diogo Freitas, Joao Botas, Bruno Mataloto, Ricardo Bezerra, Manuel Dias

**Affiliations:** 1 Information Sciences, Technologies and Architecture Research Center (ISTAR-IUL), Instituto Universitário de Lisboa (ISCTE-IUL), 1649-026 Lisbon, Portugal; 2 Faculty of Logistics, Molde University College, 6410 Molde, Norway; 3 INOV—Instituto de Engenharia de Sistemas e Computadores Inovação, 1000 Lisbon, Portugal; 4 BioGHP, 1000 Lisbon, Portugal

**Keywords:** blockchain, access control, smart contracts, decentralized identifiers, healthcare Internet of Things, consent management, permissioned distributed ledger, HL7 FHIR, audit logging, threat model

## Abstract

Health data generated by Internet of Things (IoT) devices outside institutional boundaries—consumer wearables and home-based clinical monitors—expands the attack surface for unauthorized access and complicates patient-side control over data sharing, a problem that conventional, centrally gated Electronic Health Record (EHR) architectures do not solve. Regulations such as the GDPR and the forthcoming European Health Data Space (EHDS) increasingly require auditable, revocable, patient-controlled access. This paper presents IoT2Wallets, a permissioned blockchain access-control and consent-auditing architecture combining decentralized identifiers (DIDs), W3C verifiable credentials, and two Solidity smart contracts (ConsentManager and AccessLog) on a four-node Hyperledger Besu network running IBFT 2.0 consensus (f = 1 of 4 nodes). We define a STRIDE-organized threat model describing the smart-contract-enforced mitigations in place and the threats left unaddressed. The architecture was implemented and evaluated across patient-controlled and institution-controlled consent scenarios, integrating HL7 FHIR R4 clinical and wearable data from a Parkinson’s disease remote-monitoring pilot (18 patients, 20 healthcare professional evaluators). Across 200 test transactions per operation, verifiable credential verification averaged 95 (SD 12) ms, and end-to-end access logging latency averaged 3840 (SD 490) ms, with 0/200 observed failures across consent-verification and revocation tests (Wilson 95% CI upper bound ≈ 1.8%) and complete access log fidelity relative to the independently maintained gateway request log. We discuss the scope and limits of the security guarantees established—access control and auditability rather than comprehensive cryptographic protection, formal verification, or independent smart-contract audit—and outline the hardening work required before production deployment. These results are contingent on the current implementation, which has undergone internal code review only; no independent smart-contract audit or formal verification has yet been performed, and this absence should be read as the primary limit on the security claims that follow, not a minor caveat. In addition, all four ledger nodes in the pilot were operated by the research team, so the pilot does not test the multi-institution trust argument for using a blockchain rather than a signed log.

## 1. Introduction

Health data increasingly originates outside the institutional perimeter that conventional access-control architectures are designed to protect. Consumer wearables and home-based clinical monitors generate continuous physiological and behavioral data that must eventually reach institutional systems, but the path between device and institution is rarely subject to the same access-control rigor as data that originates inside the hospital network [1,2,3,4,5,6,7]. Centralized Electronic Health Record (EHR) gateways typically mediate this path through a single trusted intermediary, which concentrates risk: a compromised or misconfigured gateway, or an institution acting unilaterally, can grant, deny, or silently alter access without an independently verifiable record, and the patient—who generated the data—has no cryptographic mechanism to enforce or audit the terms under which it is shared [8,9,10,11]. Regulatory instruments including the GDPR and the forthcoming EHDS increasingly codify a requirement that this should not be the case: patients must retain meaningful, auditable, revocable control over health data access [12,13,14,15,16,17], which is fundamentally an access-control and audit-logging requirement, not merely a data management one.

Distributed ledger technology has been proposed repeatedly as a way to decentralize this trust relationship, removing any single party’s unilateral ability to alter an access or consent record [18]. However, much of the existing literature on blockchain-based health data access control either evaluates proposed designs only in simulation, omits a systematic threat model, or does not integrate with the semantic interoperability standards (notably HL7 FHIR) that institutional systems require for the access-controlled data to be usable once released [19,20,21,22,23,24]. This leaves an open engineering question: can a permissioned blockchain access-control layer be built that (a) is grounded in a structured, STRIDE-organized, auditable threat model; (b) integrates natively with FHIR-based clinical data; and (c) is evaluated not only for latency but also for the correctness of its consent-enforcement behavior in a real clinical pilot rather than in simulation alone?

This paper addresses that question directly. We present IoT2Wallets, an access-control and consent-auditing architecture combining decentralized identifiers, verifiable credentials, and two purpose-built smart contracts on a permissioned Hyperledger Besu network, and we evaluate it against a STRIDE-organized threat model (Section 2.8) and a clinical pilot integrating wearable and institutional health IoT data for Parkinson’s disease (PD) monitoring. Our contributions are threefold: (1) an access-control architecture in which patient-controlled and institution-controlled consent governance share a common smart-contract and audit-logging core; (2) a threat model, organized around the STRIDE framework, that makes explicit both the threats mitigated by the current design and those left unaddressed; and (3) an empirical evaluation of consent-verification correctness, access logging fidelity, and latency across 200 test transactions per operation in a real clinical deployment.

### Related Work

Prior blockchain-based health data access-control proposals cluster into three groups; broader design science, trust, federated learning, and blockchain health studies frame the surrounding design space [25,26,27,28,29]. A first group evaluates access-control smart contracts against simulated or synthetic data without a clinical deployment [19,21]. A second group implements a working system but does not adopt a semantic interoperability standard such as FHIR, which limits the released data’s usability by downstream institutional systems [20,23,24]. A third group targets storage efficiency (e.g., off-chain storage via IPFS) rather than the access-control and consent-governance question addressed here [22]. Across all three groups, we did not find a system that reports a systematic, named threat model framework (e.g., STRIDE) alongside its mitigations; threats are typically discussed narratively, if at all. Table 1 positions IoT2Wallets against six representative prior systems along dimensions specifically relevant to access control: identity model, smart-contract usage, consensus and fault model, presence of a formal threat model, whether an independent security audit is reported, and patient consent granularity.

As Table 1 makes explicit, no system in this comparison—including IoT2Wallets—reports an independent security audit; this is a limitation we return to in Section 5 rather than a point of differentiation. IoT2Wallets’ contribution relative to this comparison set is the combination of a DID-anchored identity model, a STRIDE-organized threat model, and FHIR-based clinical integration within a single, clinically piloted system, not a claim of superior security assurance in an absolute sense.

## 2. Materials and Methods

### 2.1. System Overview

**Figure 1:**
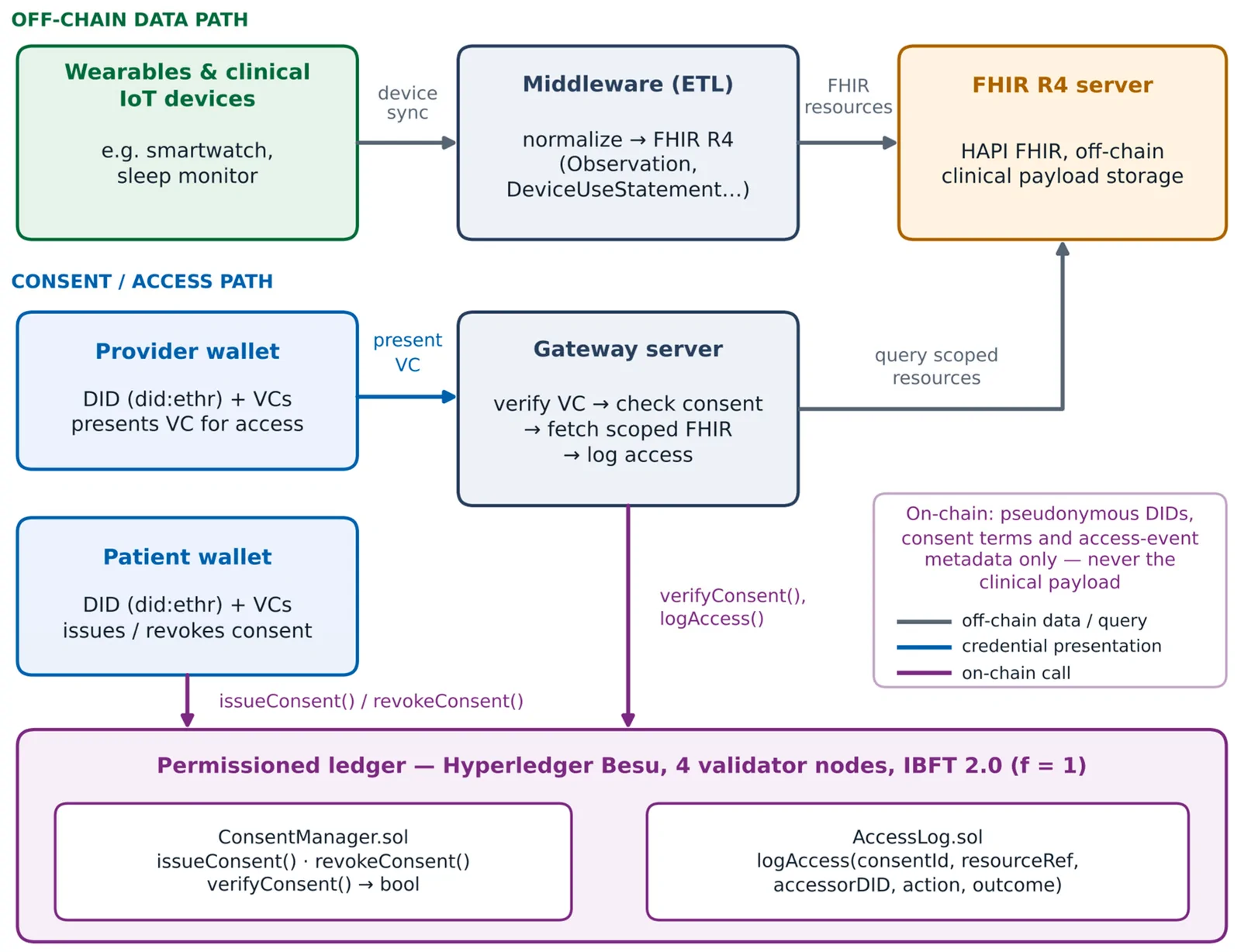
IoT2Wallets system architecture and data/consent flow. Top row: off-chain data path (devices → middleware → FHIR R4 store). Middle and bottom: consent/access path, in which the patient wallet issues or revokes consent and the provider wallet presents a verifiable credential (VC) to the gateway, which verifies consent and logs each access through ConsentManager.sol and AccessLog.sol on the four-node permissioned Hyperledger Besu ledger. Only pseudonymous DIDs, consent terms and access-event metadata are stored on-chain; clinical payloads remain off-chain.

IoT2Wallets integrates IoT sensors, a permissioned distributed ledger, and DID-based digital wallets to provide patient- and institution-controlled, auditable access to health data. Patients and healthcare professionals each hold a digital wallet acting as their identity and credential store; wearables and clinical devices synchronize data to a middleware layer that normalizes it into HL7 FHIR R4 resources before storage. Access to these resources is mediated entirely through smart-contract-enforced consent verification, described in the following subsections. Figure 1 shows the resulting architecture and the data/consent flow across these components.

### 2.2. Identity Layer

Digital wallets act as the core identity and access management tool, allowing patients and professionals to authenticate using decentralized identifiers rather than a centralized identity provider. IoT2Wallets uses the did:ethr DID method (Ethereum-based, EIP-1056 [30]), with DIDs anchored to the permissioned Hyperledger Besu chain described in Section 2.4. Concretely, each wallet holds an asymmetric key pair whose public key anchors the did:ethr identifier via the EIP-1056 registry contract, together with a local credential store holding the wallet owner’s issued verifiable credentials as signed JSON-LD; the wallet’s DID document—the public keys and service endpoints associated with the identifier—is derived from this on-chain registry entry rather than issued by a centralized identity provider. Wallets issue verifiable credentials (VCs) conforming to the W3C VC Data Model v1.1 [31], transported as JSON-LD tokens. A verifiable credential is a tamper-evident, cryptographically signed digital claim issued by one party (the issuer), held by the subject in their wallet, and presented to a verifier who can check the issuer’s signature and the credential’s validity or expiry without contacting the issuer at presentation time; unlike a plain digital certificate, its claims are selectively disclosable, independently revocable, and portable across systems via a common data model rather than a proprietary token tied to one identity provider. European digital credential and blockchain service initiatives provide relevant deployment context for this wallet model [32,33,34]. Patients use wallets to establish data sharing agreements via smart contracts, while professionals present VCs for access verification at the gateway. Operationally, the two wallet roles differ in how they interact with the system: the patient wallet signs and submits ConsentManager.issueConsent()/revokeConsent() transactions directly to the ledger, while the provider wallet does not submit a transaction at all—it presents a VC to the gateway, which itself calls the read-only ConsentManager.verifyConsent() (Section 2.5).

HL7 FHIR R4 (Fast Healthcare Interoperability Resources, Release 4) is the current global baseline for healthcare data exchange, referenced in relevant interoperability and regulatory frameworks, and defines a large catalogue of standardized resource types spanning clinical, administrative, diagnostic, medication, financial, and workflow categories, exposed through a RESTful API [35]. This is what allows the gateway server (Section 2.5) to query the off-chain FHIR store described in Section 2.4 in a standardized way rather than through a bespoke schema specific to this system, and it is consistent with patterns observed in other digital health wallet deployments [36].

### 2.3. Smart Contracts

IoT2Wallets deploys two Solidity smart contracts on Hyperledger Besu. ConsentManager.sol governs the consent lifecycle, exposing issueConsent(patientDID, providerDID, resourceRefs[], expiry, scope), revokeConsent(consentId), and verifyConsent(consentId, providerDID) → bool. All state-changing calls emit a timestamped ConsentEvent log carrying the actor’s DID, satisfying the EHDS Annex II traceability requirement [16] by construction rather than as an add-on logging layer. AccessLog.sol records every data access immutably via logAccess(consentId, resourceRef, accessorDID, action, outcome); access logs are append-only and queryable by authorized parties for audit.

Both contracts are implemented in Solidity 0.8.x and have undergone internal code review only; neither has been subjected to an independent, formal security audit at the time of writing. Given the well-documented history of exploitable vulnerabilities in deployed smart contracts across the broader blockchain literature, we treat a third-party audit as a prerequisite for production deployment rather than an optional hardening step, and we return to this explicitly in Section 5.

### 2.4. Permissioned Ledger and Consensus

Before describing the specific consensus configuration used here, we distinguish two facts this design depends on. First, a permissioned (consortium) distributed ledger restricts who may run a node or participate in consensus: validator identity is known in advance, and membership is governed by an explicit admission mechanism, in contrast to a public/permissionless chain, where participation is open and consensus is secured by economic incentives (e.g., proof-of-work or proof-of-stake) rather than by vetting participants. This distinction is what the trust argument in Section 4.2 depends on. Second, Hyperledger Besu is an Apache-2.0, enterprise-oriented Ethereum client: it is EVM- and Solidity-compatible, so contract code and tooling written for public Ethereum port to it directly, but it additionally supports consensus protocols unavailable on public Ethereum (IBFT 2.0, QBFT, Clique) and private-transaction features. Relative to deploying the same contracts on public Ethereum, Besu with IBFT 2.0 offers deterministic, immediate finality—a block committed by two-thirds of validators cannot later be reverted, unlike the probabilistic finality and reorganization risk of a proof-of-work or proof-of-stake public chain, which matters directly for a ledger whose purpose is a tamper-evident audit log. It also ensures no gas market congestion or fee unpredictability, because the network is closed to its admitted validator nodes rather than competing with unrelated public traffic, and network-level permissioning (validator/enode allowlisting) so that only admitted validators can ever propose or validate a block, which, when those validators are held by independent institutions, is the mechanism underlying the claim that no single institution can act unilaterally (Section 4.2).

The deployment used four Hyperledger Besu validator nodes with separate validator keys and IBFT 2.0 consensus, a variant of practical Byzantine fault tolerance adapted for permissioned enterprise ledgers. Node admission was controlled through network permissioning. In this pilot, all four validator nodes and their validator keys were operated by the research team; no node was held by an independent institution, and no inter-institutional governance agreement (for example, a consortium agreement defining validator admission, key custody, and removal) was in place. The pilot therefore exercised the consensus and permissioning mechanism but not the organizational independence on which the trust argument in Section 4.2 rests. That independence remains a deployment precondition that this study did not demonstrate, and we return to it as a limitation in Section 4.4. This four-node deployment tolerates up to f = 1 Byzantine (arbitrarily faulty or malicious) node without compromising ledger safety or liveness. Mechanically, validators rotate through a round-based proposer role: in each round, the current proposer broadcasts a PRE-PREPARE block proposal; validators exchange PREPARE messages; and, once a validator collects 2f + 1 matching PREPARE messages, it broadcasts COMMIT. Once 2f + 1 matching COMMIT messages are collected, the block is finalized immediately, with no forks. If a proposer fails or misbehaves, a round-change sub-protocol elects the next proposer after a timeout, preserving liveness as long as at least 2f + 1 = 3 of the 4 nodes remain honest and responsive. Each node runs (1) a ledger node holding only verifiable metadata—EHR indexes, access logs, consent terms—never patient data directly, and (2) a local FHIR R4 server (HAPI FHIR) holding the health records themselves off-chain. This off-chain/on-chain split is a deliberate design choice: it keeps clinical payloads out of the immutable ledger, which matters directly for the GDPR Article 17 erasure discussion in Section 2.6 [37], while still anchoring every access decision to an immutable, DID-attributed event.

### 2.5. Middleware and Data Flow

The middleware performs extract–transform–load (ETL) operations, normalizing device output into FHIR R4 resources (Observation, DeviceUseStatement, ActivitySummary, SleepStudyReport), validating data quality, and routing records to the FHIR server. The middleware operates purely on this off-chain data path (device → FHIR store) and has no direct interaction with the smart contracts in Section 2.3; it is a distinct component from the gateway server described next, which separately handles the on-chain consent-verification and access logging path (see Figure 1). The gateway server enforces smart-contract-based access control, verifies VCs on every request, and calls AccessLog.logAccess() on each data release. A complete access cycle proceeds in four steps. The first step is consent issuance: the patient’s wallet calls ConsentManager.issueConsent() with a defined scope and expiry, confirmed on-chain in approximately 1.8 s. The second is the access request: the clinician’s wallet presents a VC to the gateway, which calls ConsentManager.verifyConsent() (approximately 95 ms). Because this call gates every subsequent data release, its latency sits directly on the clinical interaction’s critical path, which is why it is implemented as a local read against contract state rather than a fresh on-chain transaction. The third step is data release: if consent is valid and unexpired, the gateway queries the FHIR server for the scoped resources and logs the access via AccessLog.logAccess() (approximately 1.76 s). The fourth step is revocation: the patient calls ConsentManager.revokeConsent(); all subsequent verifyConsent() calls for that identifier return false, and a ConsentRevoked event invalidates any cached data at the clinician’s application. This immediate propagation is what prevents a revoked consent from leaving a window of continued access, which is the property that Section 3.1’s revocation correctness results provide evidence for.

### 2.6. Data Governance and Erasure Reconciliation

A specific design question for any append-only, blockchain-anchored access-control system operating on personal data is how it reconciles with the GDPR Article 17’s right to erasure. IoT2Wallets is designed to substantially reduce, though not eliminate, this tension, rather than relying on exception handling logic: the blockchain stores only consent terms, resource references, and access-event metadata keyed to pseudonymous DIDs, while the underlying FHIR clinical payload always resides off-chain in the HAPI FHIR server. Erasure of a patient’s clinical data is therefore satisfied by deleting or de-identifying the off-chain FHIR resource; the on-chain log continues to reference it only by an opaque identifier and is retained after erasure, consistent with the EHDS Annex II requirement for durable audit evidence, analogous to the retention of financial or clinical audit trails after the underlying record has been deleted elsewhere.

This reconciliation resolves the erasure of the clinical payload itself, but it does not settle a separate and currently unresolved question: whether the pseudonymous on-chain identifiers and access-event metadata that remain on the ledger after erasure are themselves “personal data” under GDPR Article 4(1) [37], given that a patient in a small, longitudinal, diagnosis-linked cohort could plausibly be re-identified from that metadata—the same re-identification risk this paper invokes elsewhere to justify not publishing the raw wearable dataset. The relevant guidance points in both directions. The European Data Protection Board’s guidelines on blockchain processing state that public keys and similar on-chain identifiers qualify as personal data where the users are natural persons who can be identified by means reasonably likely to be used—for example, after a data breach—and that replacing identifiers with cryptographic or pseudonymous values does not by itself remove them from the scope of the GDPR [38]. The Court of Justice of the European Union has held that whether data are personal depends on the means reasonably likely to be used to identify the person [39] and, more recently, that pseudonymized data may fall outside that definition for a recipient who cannot reasonably re-identify the data subjects yet remains personal data for the controller that holds the means of re-identification [40]. Because the institutions operating an IoT2Wallets deployment would hold the mapping between DIDs and patients, the on-chain records are most plausibly personal data at least for those institutions. We treat this as an open legal question rather than one this architecture resolves and flag it explicitly in Section 4.4 as a limitation on the GDPR/EHDS compliance motivation framed in the Introduction.

Compared with the content-addressed IPFS model described by Jayabalan and Jeyanthi [22], the evaluated deployment used an institutionally hosted FHIR R4 server as its off-chain store. Within this architecture, the server remained under institutional operational and legal control, supported record-level deletion, and exposed the structured query model needed for scoped clinical requests. These properties align with the erasure approach evaluated here; this study did not experimentally compare FHIR server and IPFS storage models.

### 2.7. Deployment Scenarios

Two deployment scenarios were implemented and evaluated. Scenario 1 (patient-controlled, external FHIR) has consumer wearables synchronize data to the patient’s mobile wallet; measurements are normalized to FHIR R4 resources and stored off-chain in an external FHIR server, with consent issued, scoped, and revoked by the patient via ConsentManager.sol. This model targets cross-institutional, patient-sovereign data sharing consistent with the EHDS. Scenario 2 (institution-controlled, internal FHIR) has the same wallet and blockchain mechanisms interface with institutional endpoints, but access is governed by role-based smart contracts referencing institutional credential registries rather than patient wallets, which accommodates institutions operating within national EHR systems that require interoperable IoT extensions. Both scenarios share the identity layer, smart contracts, and middleware described above; they differ specifically in who controls the consent issuance step, which is the architectural property that this paper’s evaluation is designed to test.

### 2.8. Threat Model and Security Analysis

We organize the threat model using the STRIDE framework (Spoofing, Tampering, Repudiation, Information Disclosure, Denial of Service, Elevation of Privilege), a widely used taxonomy for structuring security analysis of networked systems. For each category, Table 2 states the specific threat instance relevant to IoT2Wallets, the architectural mitigation in place, and—where a category is not, or only partially, addressed—an explicit statement of that gap rather than a claim of coverage. This analysis is a system-level, single-instance-per-category mapping rather than a full per-component, trust-boundary decomposition; the latter—enumerating threats separately for each architectural component (wallet, gateway, middleware, FHIR server, ledger node) and the trust boundaries between them—is identified as future work in Section 5. We adopt this explicit-gap convention deliberately: a threat model that only lists mitigated threats is less useful, and less honest, than one that also states what has not yet been tested or resolved.

Three points about Table 2 warrant emphasis. First, the Spoofing, Tampering, Repudiation, and Elevation of Privilege categories are addressed by mechanisms that were exercised empirically in this study—consent-verification correctness (Section 3), append-only log integrity, and role-based access-enforcement testing across 60 test cases—and are reported as “implemented and tested” on that basis, not on the basis of formal proof. No formal verification (e.g., model checking or a machine-checked security proof of the ConsentManager or AccessLog contracts) was performed, and we do not claim one. Second, Information Disclosure is only partially addressed: consent-gated access control is in place and tested, but transport- and storage-level encryption is not yet uniformly enforced across all system components, which is precisely the boundary that the phrase “access control and auditability, not comprehensive data protection” in the Abstract is intended to convey. Third, Denial of Service is explicitly out of scope for the present evaluation; IBFT 2.0 provides Byzantine fault tolerance for ledger consistency under up to one faulty node in the four-node cluster used here, but no request-flooding, resource-exhaustion, or availability-focused adversarial testing was conducted against the gateway, middleware, or FHIR server.

This threat model is deliberately scoped to the access-control and audit-logging layer that is this paper’s primary contribution. It does not cover, and should not be read as covering, wallet private-key compromise or loss (which would require a separate key-recovery and revocation mechanism, currently left to future work), side-channel attacks against the underlying Hyperledger Besu or Solidity execution environment (which are the subject of a substantial existing literature on smart-contract security that this paper does not attempt to reproduce or extend), or adversarial manipulation of the wearable or clinical devices themselves prior to data reaching the middleware. We consider each of these a legitimate direction for follow-on security research building on the architecture presented here, rather than a gap that this paper resolves.

### 2.9. Experimental Testbed, Execution Environment, and Measurement Protocol

All reported performance and correctness results were produced on a controlled institutional testbed, not on a public blockchain or a live hospital production network. The testbed was hosted on ISCTE-IUL infrastructure and was reachable only from the institutional network. It comprised four Hyperledger Besu validator nodes running IBFT 2.0 consensus; each node ran Ubuntu 22.04 on an 8-core CPU with 16 GB RAM and had a separate validator key. Node admission was controlled through network permissioning. No clinical payload was written to the ledger: on-chain records contained consent terms, opaque resource references, and access-event metadata, while clinical resources remained in a HAPI FHIR R4 server off-chain.

The software stack comprised Hyperledger Besu, a HAPI FHIR R4 server, Solidity 0.8.x smart contracts, the did:ethr DID method, and W3C verifiable credentials represented as signed JSON-LD. The patient wallet and clinician-facing gateway interfaces used HTTPS (TLS). Internal traffic between the gateway, middleware, and FHIR server remained on the testbed’s internal network and was not TLS-protected; the FHIR data store was not encrypted at rest. These controls and gaps are material to interpreting the pilot as an access-control evaluation rather than an end-to-end confidentiality evaluation.

All four validator nodes and their validator keys were operated by the research team. The testbed therefore exercised permissioned consensus, consent enforcement, revocation, and audit logging but did not test a multi-institution governance model or public-network performance. Administrative access was limited to the institutional network and research team members responsible for the deployment.

For each operation in Table 3, 200 test transactions were executed on this testbed. The measured operations were ingestion of a single FHIR observation, on-chain consent confirmation, access log writing, verifiable credential verification, and the end-to-end device-to-FHIR-to-ledger path. Table 3 reports the mean, standard deviation, minimum, and maximum latency for these 200 observations. The correctness evaluation separately comprised 200 consent-verification and revocation tests across both scenarios and 60 role-based access tests in Scenario 2.

The manuscript record does not specify host model or storage media; exact Besu, HAPI FHIR, Java, Node.js, and Solidity compiler builds; genesis and block-period settings; endpoint configuration; or network bandwidth and latency. Those parameters are necessary to reproduce absolute latency values. Accordingly, the results in Section 3 are reported as implementation-specific testbed measurements and not as deployment-independent performance claims.

### 2.10. Clinical Pilot

The architecture was evaluated in a pilot integrating data from 18 patients with PD (11 male, 7 female; mean age 64.3, SD 7.8 years; Hoehn and Yahr stages I–III), each wearing a clinical sleep and activity monitor together with a consumer smartwatch (Garmin Vívosmart 5, firmware 9.90; Garmin Ltd., Olathe, KS, USA) for 12 consecutive nights, which yielded 216 patient-nights of paired device data. Access-control usability was evaluated with 20 healthcare professionals (8 neurologists, 7 nurses, 5 health informatics specialists) via the System Usability Scale (SUS) after a structured demonstration of the wallet and consent management interface. All procedures were approved by the Institutional Ethics Committee of Instituto Universitário de Lisboa (ISCTE-IUL; reference IUL-2023-ETH-0047; approved on 17 February 2023), with written informed consent obtained from all participants.

Regarding protection of patient data during the pilot, the controls in place while the pilot handled data from the 18 patients were as follows. (i) Transport: The external interfaces, from the patient wallet to the gateway and from the clinician application to the gateway, were served over HTTPS (TLS). Internal traffic between the gateway, the middleware, and the FHIR server ran over the testbed’s internal network without TLS. (ii) Storage: The HAPI FHIR server’s data store was not encrypted at rest. (iii) Ledger content: No clinical payload was written to the ledger; on-chain records held only consent terms, opaque resource references, and access-event metadata keyed to pseudonymous DIDs (Section 2.6). (iv) Release control: Every release of a FHIR resource to a clinician application passed through the gateway’s verifiable credential, consent, and role checks (Section 2.5 and Section 3.1). (v) Administrative access by study staff: The testbed was hosted on ISCTE-IUL infrastructure and reachable only from the institutional network; administrative access was held by members of the research team responsible for the deployment. No study-specific access-control policy, role separation, or access logging beyond the host defaults was in place for administrative access during the pilot. Because of (i) and (ii), confidentiality of the pilot data inside the testbed depended on the internal network boundary and on administrative access control, not on cryptographic protection: a party with access to the internal network or to the FHIR host would not have been stopped by the mechanisms evaluated in this paper. The Information Disclosure entry in Table 2 should therefore be read as partial for the pilot itself, not only for the architecture in general.

### 2.11. Outcome Measures

We report three classes of outcomes relevant to an access-control and audit-logging architecture: (1) correctness of consent verification and revocation across 200 test transactions; (2) latency of FHIR ingestion, on-chain consent confirmation, access log writes, and VC verification; and (3) access log completeness, measured as the concordance between the independently maintained gateway request log and the on-chain AccessLog record. Usability (SUS) is reported as a secondary, human-factors outcome relevant to whether the access-control mechanism is operable by its intended users, not as a security metric per se.

## 3. Results

### 3.1. Consent-Verification and Revocation Correctness

All 200 consent-verification transactions returned the correct result across both deployment scenarios. (0/200 failures; Wilson 95% CI upper bound ≈ 1.8%.) Consent revocation propagated to the gateway within one block confirmation time in every test case, and no FHIR resource was released after revocation; verifiable credentials that had reached their configured expiry (30-day window in Scenario 1) were likewise denied access in all post-expiry attempts (0 failures observed). Role-based access enforcement in Scenario 2 (roles: attending physician, case manager, and research nurse) was correct in all 60 test cases, with no instance of an out-of-role request being incorrectly granted or an in-role request being incorrectly denied.

### 3.2. Latency

Table 3 reports latency across 200 test transactions per operation. All figures below were measured on the four-node Besu testbed described in Section 2.9, using data volumes and access patterns drawn from the clinical pilot; they are testbed measurements, not production latencies from a live hospital deployment.

Verifiable credential verification—the operation gating every access request—averaged 95 (SD 12) ms, well within the range acceptable for interactive clinical use; this characterization applies specifically to the 95 ms verification step and not to the longer end-to-end figure below. On-chain consent confirmation and access log writes, at approximately 1.8 s each, reflect block confirmation time under IBFT 2.0 and dominate the 3840 (SD 490) ms end-to-end figure. The gap between the mean and maximum end-to-end latency (3840 ms vs. 5210 ms) is consistent with a right-skewed distribution typical of blockchain confirmation times; a full percentile (95th/99th) characterization was not performed in this pilot and is identified in Section 5 as a specific target for a dedicated scalability evaluation, as mean and SD alone can understate the experience of slower requests under such a distribution. No transactions-per-second figure was measured, and no concurrency or load testing was performed against the gateway, middleware, or ledger; Section 5 identifies throughput and concurrency testing, alongside tail latency characterization, as a specific target for a dedicated scalability evaluation.

#### Comparability with Prior Work

The related-work analysis in Table 1 considers six representative blockchain-based health data sharing systems: Moulahi et al. [19], Zhuang et al. [20], Ghadi et al. [21], Jayabalan and Jeyanthi [22], Wu et al. [23], and MedRec [24]. They were selected because they address blockchain-supported health data sharing, access control, or related consent and storage functions. Table 1 compares the systems qualitatively on identity model, smart-contract use, consensus and fault model, threat model reporting, independent audit reporting, and consent granularity.

We do not claim a numerical cross-study latency benchmark. The comparator studies use different ledger deployments, consensus mechanisms, operations, workloads, host resources, and network conditions, and the present study did not rerun their implementations under a common protocol. The values in Table 3 are therefore an internal baseline for the specified four-node testbed. They support the feasibility of the implemented workflow but do not establish that IoT2Wallets is faster than the systems in Table 1. Table 4 makes this boundary explicit.

### 3.3. Access Log Completeness

On-chain access logs showed 100% event-capture fidelity against the independently maintained gateway request log across both deployment scenarios: every access event recorded at the application layer had a corresponding, matching entry on the permissioned ledger, and no silent logging gaps were observed across the 216 patient-nights of Scenario 1 data or the 60 role-based test cases of Scenario 2. All eight EHDS Annex II mandatory event types were captured in the access log. This end-to-end concordance between application-level and ledger-level records is the specific property the Repudiation mitigation in Table 2 depends on, and it held throughout the pilot.

### 3.4. Usability of the Access-Control Interface

The mean SUS score across the 20 healthcare professional evaluators was 71.4 (SD 8.2), corresponding to “good” usability and above the commonly cited acceptability benchmark of 68 [41] for this instrument. A descriptive, exploratory comparison suggested somewhat higher ratings among health informatics specialists (mean 76.2) than neurologists (mean 68.9); with subgroups of only five and eight respondents, respectively, this study was not powered to test whether the difference is statistically meaningful, and no inferential test was performed. Open-ended feedback identified audit log interpretability—scanning the immutable log for a specific past access—as the most frequently raised concern, consistent with audit log content, which bears on the Information Disclosure category, being harder to interpret for end users than the binary allow/deny outcome of a consent check.

## 4. Discussion

### 4.1. Principal Findings

This study shows that a permissioned blockchain access-control layer built around a STRIDE-organized threat model (a system-level, single-pass analysis—see Section 2.8) and integrated natively with HL7 FHIR can be deployed in a real clinical pilot with fast, correct consent enforcement (95 ms verification latency, 0/200 observed failures across 200 test transactions) and complete audit log fidelity. These are observed results from 200 test transactions per operation on an implementation that has undergone internal code review only. Because no independent smart-contract audit or formal verification has been performed (Section 4.4), they show that consent enforcement behaved correctly in the cases tested, not that the contracts are free of exploitable defects. The architecture supports two structurally different consent-governance models—patient-sovereign and institution-sovereign—from a single smart-contract and audit-logging core, which is the specific architectural claim motivating this work.

### 4.2. Trade-Offs of a Blockchain-Based Design

A reasonable alternative to a blockchain-based consent ledger is a simpler, centrally hosted, cryptographically signed audit log, which would avoid the on-chain confirmation latency (approximately 1.8 s per consent or access log transaction) documented in Section 3.2. We adopt a permissioned blockchain specifically because it removes the institution itself as a single party able to unilaterally alter the consent or access history—a property that a centrally hosted signed log cannot provide even if tamper-evident, because the log operator retains unilateral control over what is written in the first place. This property directly serves the Repudiation mitigation in Table 2 and the cross-institutional trust model of Scenario 1, where no single institution is the natural custodian of the audit trail. This argument holds only to the extent that the node operators are genuinely independent parties; if a single institution were to control a majority of nodes, the central advantage over a signed log would not be sustained. In the pilot reported here, this condition was not met: all four validator nodes were operated by the research team (Section 2.4). In this deployment, therefore, the ledger offered no stronger protection against the unilateral alteration of the consent or access history than a signed log held by the same team would have. The pilot supports the functional claims in Section 3 (consent enforcement, revocation, and audit log fidelity) but not the trust argument for a blockchain over a signed log. That argument remains a design rationale, to be tested in a multi-institution deployment in which each validator is held by a separate organization under a written governance agreement. We treat this independence as an explicit precondition on the trust claim, not a property that the blockchain mechanism guarantees by itself. Where all parties already trust a single institutional operator and cross-institutional portability is not required, a simpler signed log design remains a reasonable, lower-latency alternative, and we do not claim that the blockchain-based design is necessary in that narrower deployment context.

### 4.3. Comparison with Prior Work

As Table 1 shows, IoT2Wallets is, to our knowledge, the only system in this comparison set that combines a DID-anchored identity layer, an explicit STRIDE-organized threat model, and native FHIR R4 integration within a clinically piloted implementation. We note that our threat model is a system-level STRIDE mapping rather than a full per-component decomposition (Section 2.8), so this comparison should be read as a difference in reporting practice within the literature rather than a claim of superior security assurance. MedRec [24] and Wu et al. [23] implement smart-contract-based access control without a DID layer or FHIR alignment; Moulahi et al. [19] and Ghadi et al. [21] propose or evaluate designs without a clinical deployment; and none of the six comparator systems reports a named threat model framework or an independent security audit. We read this pattern as indicating that systematic, named threat modeling remains underused in this literature relative to the maturity of the underlying blockchain and access-control mechanisms being proposed, which is as much a reporting gap in the field as a property of any individual system.

### 4.4. Limitations

This study’s security evaluation has clear boundaries that qualify every claim made above. The threat model in Section 2.8 and its empirical support in Section 3 address access control, consent enforcement, and audit log integrity specifically; they do not constitute a comprehensive security evaluation of the system. Neither the ConsentManager nor the AccessLog smart contract has undergone an independent, formal security audit, and no formal verification (model checking, theorem proving, or symbolic protocol analysis) was performed on either contract; both are prerequisites we consider necessary, not optional, before production deployment, and their absence is the single most important boundary on the claims in this paper. Denial-of-service resilience was not evaluated: the gateway, middleware, and FHIR server were not subjected to request-flooding or resource-exhaustion testing, and their behavior under such conditions is unknown. Transport- and storage-level encryption is not yet uniformly enforced across all system components, so the Information Disclosure mitigation in Table 2 should be read as partial. The controls that did protect patient data during the pilot (TLS on the external wallet- and clinician-facing interfaces, gateway-mediated release, and keeping clinical payloads off-chain) are listed in Section 2.10. Internal traffic was not TLS-protected, and the FHIR store was not encrypted at rest, so these controls should not be interpreted as evidence of end-to-end confidentiality. The four-node testbed was not load-tested beyond 200 transactions per operation, and tail latency (95th/99th percentile) was not characterized, even though the right-skewed shape of the observed latency distribution suggests that the tail behavior may differ materially from the mean; continuous multi-patient streaming at hospital scale would generate substantially more on-chain writes than this pilot exercised, and the resulting throughput ceiling is unknown. Wallet private-key compromise, loss, and recovery were explicitly out of scope. Finally, the clinical pilot itself was small (18 patients, 20 evaluators), determined by clinic recruitment capacity rather than a power calculation, and sufficient to establish technical and functional feasibility but not generalizability; the full source code is currently available from the corresponding author on reasonable request. The smart contracts and the non-patient-facing gateway and middleware code will be released publicly upon acceptance (see Data Availability Statement), and the complete implementation will follow the independent audit.

Three further limitations warrant explicit emphasis. First, the trust argument in Section 4.2 for a blockchain over a simpler signed log depends on the ledger nodes being operated by genuinely independent institutions. In this pilot, all four nodes were operated by the research team (Section 2.4), so the pilot does not demonstrate that argument, and it should be read as a design rationale rather than an evaluated result. Second, whether the pseudonymous on-chain identifiers and access-event metadata retained after erasure constitute “personal data” under GDPR, given re-identification risk in a small, diagnosis-linked cohort, is an open legal question that Section 2.6’s erasure design does not settle. Third, no transactions-per-second figure was measured, and no concurrency or load testing was performed, so the system’s behavior under realistic multi-patient, hospital-scale request volume is unknown, and the “fine-grained, per-resource” consent granularity claimed in Table 1 is supported by design but was not separately evaluated in Section 3, which tested consent-level verification, revocation, and role-based access rather than resource-level granularity specifically.

## 5. Conclusions

This paper presented IoT2Wallets, a permissioned blockchain access-control and consent-auditing architecture for health IoT data built around decentralized identifiers, verifiable credentials, and two purpose-built smart contracts, and evaluated it against a STRIDE-organized threat model (a system-level, single-pass analysis) in a real clinical pilot. Across 200 test transactions per operation, the architecture achieved 0/200 observed failures in consent-verification and revocation testing, 95 (SD 12) ms verifiable credential verification latency, and complete access log fidelity against an independently maintained request log, while supporting two structurally different consent-governance models from a single smart-contract core. Twenty healthcare professionals rated the resulting interface as having good usability (SUS 71.4). These results were obtained on contracts that have undergone internal code review only. Neither contract has been independently audited or formally verified, which is the most important boundary on the claims above. In addition, all four ledger nodes were operated by the research team, so the multi-institution trust argument for a blockchain over a signed log was not tested.

These findings are bounded to the documented four-node institutional testbed. They are not an external performance benchmark, do not characterize concurrency or hospital-scale load, and cannot be generalized to a deployment with different software builds, network conditions, hardware, or governance arrangements.

These results establish the technical and functional feasibility of the access-control architecture and make explicit, via Table 2, precisely which threats it currently mitigates and which it does not. They do not, and are not presented as, a comprehensive security validation. The specific, prioritized next steps we consider necessary before production clinical deployment are: (1) an independent, third-party security audit of both the ConsentManager and AccessLog smart contracts; (2) formal verification of the consent lifecycle (issuance, verification, revocation, expiry) against the STRIDE threats identified in Table 2, ideally via a symbolic protocol-analysis tool; (3) denial-of-service and resource-exhaustion testing of the gateway, middleware, and FHIR server; (4) uniform transport- and storage-level encryption across all system components; (5) a dedicated scalability evaluation characterizing tail latency (95th/99th percentile) under a multi-patient, hospital-scale load; and (6) a wallet key-recovery mechanism addressing private-key loss or compromise. We distinguish items (1) and (2) deliberately: an independent audit brings an adversarial, specialized review to find implementation-level vulnerabilities well documented in deployed Solidity contracts generally (e.g., reentrancy, unchecked external calls, access-control bypass) that are easy for the original developers to overlook, while formal verification would instead mathematically prove or disprove specific safety properties of the consent lifecycle directly (e.g., that no credential can pass verifyConsent() after its associated revokeConsent() has been mined)—a guarantee that test coverage alone can, by construction, only support on the cases actually tested. We add three further items to this list: (7) a full per-component, trust-boundary STRIDE decomposition (Section 2.8) rather than the system-level, single-instance-per-category mapping presented here; (8) a throughput (transactions-per-second) and concurrency/load evaluation of the gateway, middleware, and ledger under realistic multi-patient request volume, alongside the tail latency characterization already listed; and (9) an evaluation isolating resource-level consent granularity specifically, rather than the consent-level verification, revocation, and role-based access tested in Section 3. We regard the architecture and threat model presented here as a sound and clinically piloted foundation for this work and as defining a concrete, falsifiable set of next steps rather than a completed security evaluation.

## Figures and Tables

**Table 1:** Security-relevant comparison of IoT2Wallets with related blockchain-based health data access-control systems.

System	DID/SSI	Smart Contracts	Consensus/Fault Model	Formal Threat Model	Independent Security Audit	Patient Consent Granularity
IoT2Wallets (this study)	Yes (did:ethr)	Yes (2 contracts)	IBFT 2.0, f = 1 of 4 nodes	Yes (this paper)	Not performed (see Limitations)	Fine-grained, per-resource, wallet-mediated (supported by design; not separately evaluated—see Section 4.4)
MedRec (Azaria et al.) [24]	No	Yes	PoW (public Ethereum)	Partial (informal)	Not reported	Provider-mediated
Zhuang et al. [20]	No	Yes	PoW (private Ethereum)	Not reported	Not reported	Patient-controlled, coarse
Moulahi et al. [19]	No	No (federated learning only)	Permissioned, unspecified	Not reported	Not reported	Federated (no per-patient wallet)
Ghadi et al. [21]	No	Partial	Public/private, unspecified	Not reported	Not reported	Institutional only
Wu et al. [23]	No	Yes (re-encryption)	Consortium, unspecified	Not reported	Not reported	Patient audit trail only
Jayabalan and Jeyanthi [22]	No	Yes	Permissioned, unspecified	Not reported	Not reported	Institutional only

Note in Table 1: “Not reported” indicates that the cited publication does not describe the property in question, not that the comparator system necessarily lacks it. This assessment reflects the authors’ reading of the cited papers and may be incomplete.

**Table 2:** STRIDE-organized threat model for IoT2Wallets.

STRIDE Category	Threat Instance in IoT2Wallets	Mitigation	Status
Spoofing	An unauthorized party presents a forged or stolen identity to request patient data.	Gateway authentication via W3C verifiable credentials tied to did:ethr identifiers; every request is checked against ConsentManager.verifyConsent().	Implemented and tested
Tampering	A party attempts to alter a previously issued consent record or an access log entry after the fact.	Consent state is held in ConsentManager.sol; access events are written to the append-only AccessLog.sol. Both benefit from IBFT 2.0 immutability, tolerating up to f = 1 faulty node in the 4-node cluster used here.	Implemented and tested
Repudiation	A patient, provider, or institution denies having issued, accessed, or revoked a consent.	Every ConsentManager and AccessLog call emits a timestamped, DID-attributed event (ConsentEvent, AccessEvent), satisfying EHDS Annex II traceability and providing non-repudiation of both the action and the actor.	Implemented and tested
Information Disclosure	Data at rest or in transit is intercepted or read by a party without valid consent.	Access to FHIR resources is gated by smart-contract consent verification. Transport- and storage-level encryption is not yet uniformly enforced across all components in the current prototype.	Partially addressed—see Limitations
Denial of Service	A party floods the gateway, FHIR server, or blockchain nodes with requests to degrade availability.	Not addressed by the current architecture. IBFT 2.0’s Byzantine fault tolerance protects ledger consistency under node failure or malicious behavior but was not evaluated against request-flooding or resource-exhaustion attacks.	Out of scope—not evaluated
Elevation of Privilege	A party with a valid but narrower-scoped credential attempts to access data or actions outside that scope (e.g., a nurse role attempting physician-only actions).	Role- and scope-bound verifiable credentials checked against consent scope and role registries in ConsentManager.sol before any AccessLog.logAccess() call is permitted.	Implemented and tested (60 role-based test cases)

**Table 3:** System latency measurements (200 transactions per operation).

Operation	Mean (ms)	SD (ms)	Min (ms)	Max (ms)
FHIR resource ingestion (single observation)	142	18	118	201
On-chain consent transaction confirmation	1840	310	1440	2620
Access log write (EHDS Annex II event)	1760	280	1390	2510
Verifiable credential verification (smart contract)	95	12	81	134
End-to-end (device → FHIR → on-chain log)	3840	490	3110	5210

**Table 4:** Boundary between internal testbed measurements and cross-study comparison.

Evaluation Question	Evidence in This Study	Cross-Study Comparison Status
Consent enforcement and revocation	200 consent-verification and revocation tests; 60 role-based tests.	Internal functional evidence; no common external workload was run.
Latency	200 observations for each Table 3 operation on the four-node institutional testbed.	No direct performance ranking; network, hardware, consensus, and operation definitions differ across studies.
Architecture and assurance reporting	DID, FHIR, contracts, IBFT 2.0, STRIDE mapping, and audit status.	Qualitative comparison with the six systems in Table 1.

## Data Availability

The original data presented in this study are openly available: a de-identified synthetic FHIR R4 dataset (50 clinical bundles and 600 per-night wearable observation bundles) is deposited in Zenodo at doi:10.5281/zenodo.20853127 [43]. The underlying real-patient wearable dataset (18 patients) is not publicly deposited given the indirect re-identification risk associated with a small, single-clinic cohort with a rare, identifiable diagnosis and detailed longitudinal physiological data; aggregate, cohort-level summary statistics are reported in Section 3, and a fully de-identified, aggregated extract may be made available from the corresponding author on reasonable request, subject to institutional approval. The ConsentManager.sol and AccessLog.sol smart contracts, together with the non-patient-facing gateway and middleware code, will be released in a public repository, archived with a Zenodo DOI, upon acceptance of this article; deployment credentials, network configuration, and clinic-specific integration settings will be removed from this release. The complete implementation will be released after the independent audit described in Section 5. Until then, the full code is available from the corresponding author on reasonable request.
